# Acupuncture at Cuanzhu (BL2) for persistent hiccups following laryngeal mask airway insertion under general anesthesia: A case series

**DOI:** 10.1097/MD.0000000000045556

**Published:** 2025-11-21

**Authors:** Li Chen, Guo-Ao Shi, Shan Wang, Xu Dong, Chun-Ru Zhang, Li Kang, Dong-Sen Lv

**Affiliations:** a Shenzhen Bao’an Traditional Chinese Medicine Hospital, Shenzhen, Guangdong Province, China; b Shenzhen Longgang People’s Hospital, Shenzhen, Guangdong Province, China.

**Keywords:** acupuncture, case series, Cuanzhu (BL2), intraoperative hiccups

## Abstract

**Rationale:**

Pharmacological management of intraoperative hiccups under general anesthesia is often limited by adverse effects. This series evaluates BL2 acupuncture as a nonpharmacological alternative for persistent hiccups when pharmacotherapy is contraindicated.

**Patient concerns:**

Five patients (27–46 years) developed persistent hiccups following laryngeal mask airway insertion, compromising surgical safety.

**Diagnoses:**

Persistent intraoperative hiccups refractory to conventional management.

**Interventions:**

Bilateral BL2 acupuncture using standardized manipulation techniques.

**Outcomes:**

Hiccups ceased within 4–8 minutes (median: 6; interquartile range [IQR]: 4.5–7.0). One recurrence occurred at 30 minutes postoperatively. No adverse events were recorded. Minimal fluctuations in bispectral index (median: 3; IQR: 2.0–4.5) and mean arterial pressure (median: 4; IQR: 3.5–4.5) were observed.

**Lessons:**

BL2 acupuncture offers a technically straightforward, effective intervention for intraoperative hiccups with preserved hemodynamic stability, serving as a viable nonpharmacological alternative when conventional medications are unsuitable.

## 1. Introduction

Intraoperative hiccups, characterized by involuntary diaphragmatic contractions, represent an uncommon yet clinically significant complication during general anesthesia (GA). They may compromise the stability of the surgical field, impair the precision of surgical maneuvers, and adversely affect respiratory function by altering pulmonary compliance.^[1]^ Furthermore, hiccups can disrupt the function of the lower esophageal sphincter, heightening concern about perioperative aspiration risk.^[2]^

There are no established treatment guidelines for hiccups. Non-perioperative hiccups primarily rely on pharmacological treatments such as chlorpromazine, metoclopramide, sedatives, and proton pump inhibitors. However, these medications are associated with adverse effects, such as hypotension, sedation, and extrapyramidal symptoms, etc, which limit their utility during intraoperative settings.^[3,4]^ For intraoperative hiccups under GA, neuromuscular blocking agents(NMBA) or deepening anesthesia are commonly employed,^[5]^ though these interventions may precipitate hemodynamic instability and delayed postoperative recovery.

Acupuncture was found to be an effective non-pharmacologic intervention.^[6]^ Acupuncture may alleviate hiccups by enhancing hypothalamic blood flow and exerting bidirectional regulatory effects on gastrointestinal motility.^[7]^ The Cuanzhu acupoint (BL2), an empirically validated point for hiccups treatment, achieved a 95.83% satisfaction rate (vs 81.25% in controls) when used as the primary intervention for refractory non-perioperative hiccups.^[8]^ However, there have been no reported cases of needling BL2 to treat intraoperative hiccups under GA. This study analyzed 5 cases of intraoperative hiccups occurring after laryngeal mask airway (LMA) insertion under GA. By applying BL2 acupuncture, we observed the termination of hiccups, the number of recurrence cases within 24 hours, local adverse reactions at acupuncture sites, and fluctuations in bispectral index (BIS) and mean arterial pressure (MAP) before intervention and after hiccups cessation to evaluate its applicability in intraoperative settings.

## 2. Methods

### 2.1. Clinical data

In this study, intraoperative persistent hiccups were defined as hiccups occurring intraoperatively, characterized by a frequency of ≥3 episodes per minute and lasting >3 minutes. We report 5 consecutive cases (American Society of Anesthesiologists physical status class I-II, aged 27–46 years, 3 females and 2 males) who developed persistent hiccups after LMA insertion under GA at Shenzhen Bao’an Traditional Chinese Medicine Hospital between September 2024 and April 2025. The research protocol was approved by the Medical Ethics Committee of Shenzhen Bao’an Traditional Chinese Medicine Hospital (Ethics Approval No.: KY-2025-013-01). As the data were anonymized, the requirement for informed consent was waived.

### 2.2. Treatment protocol

**Figure 1.**
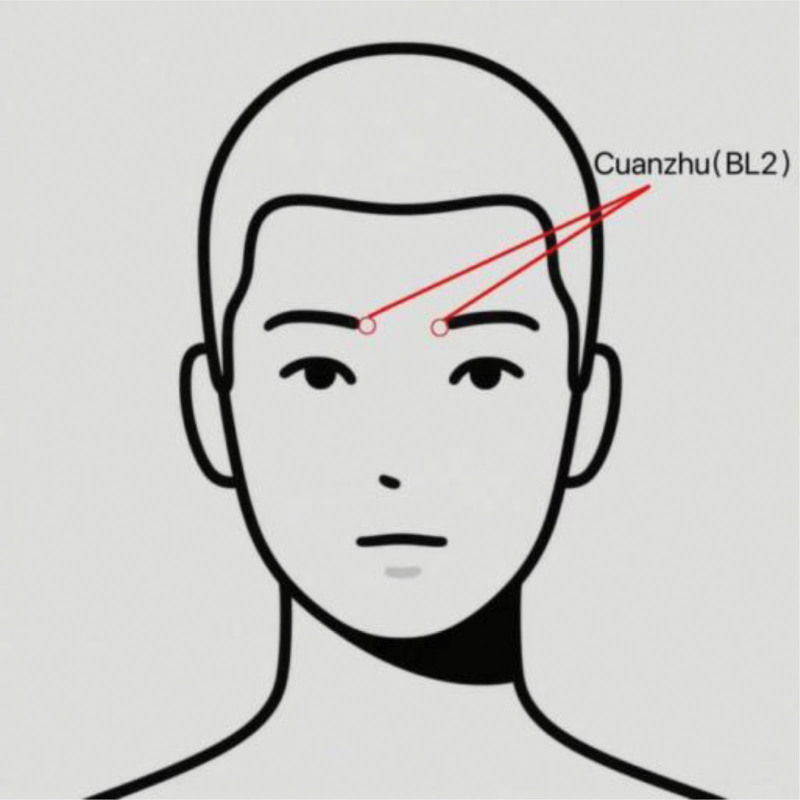
Anatomical location of the Cuanzhu (BL2) acupoint. The point is located on the medial end of the eyebrow, in the supraorbital notch.

Following unsuccessful conventional physical interventions (e.g., adjusting LMA position, hyperextending the neck) for intraoperative hiccups, acupuncture at the BL2 was performed by a licensed acupuncturist with over 3 years of clinical experience. BL2 was localized according to World Health Organization standard acupoint definitions: at the medial end of the eyebrow, in the depression superior to the supraorbital notch (Fig. 1).

After disinfecting the bilateral BL2 regions with 75% alcohol, disposable sterile stainless steel acupuncture needles (0.25 mm × 25 mm; Suzhou Medical Supplies Co., Ltd., China) were inserted obliquely downward toward the orbit at a 10° to 20° angle relative to the skin surface, inserting at a depth of 5 to 10mm. After needle insertion, rotary manipulation (rotating the needle shaft 0.3–0.5cm in amplitude at 60 times.min^−1^) and lifting-thrusting manipulation (vertically moving the needle 0.5–1cm in amplitude at the same frequency) were alternately performed using the thumb and index finger gripping the needle handle. Both techniques maintained linear alignment of the needle body. Each combined rotary and lifting-thrusting session lasted 1 minute and was repeated every 5 minutes until hiccups cessation. The needles were retained until the conclusion of surgery and removed upon exiting the operating room.

### 2.3. Outcome measures

#### 2.3.1. Primary outcomes

Hiccups cessation time: Defined as the time interval (in minutes) from acupuncture initiation to complete cessation of hiccups. Symptom relief and cessation were assessed by both the attending anesthesiologist and operating room nurse.

#### 2.3.2. Secondary outcomes

Hiccup recurrence: Included the number of recurrent cases, frequency of postoperative episodes, and cumulative duration of hiccups within 24 hours postoperatively.

#### 2.3.3. Safety outcomes

Local indicators: pain, hemorrhage, or ecchymosis at the acupuncture site. Pain intensity was evaluated using the visual analogue scale (VAS).

Systemic indicators: fluctuations in BIS and MAP before acupuncture and after hiccups cessation. Significant fluctuations were defined as: BIS variation ≥ 10, or MAP variation ≥ 20 mm Hg.

#### 2.3.4. Efficacy evaluation criteria

Complete remission: Hiccups ceased entirely with no recurrence within 24 hours.

Partial remission: occasional hiccups posttreatment or ≥50% reduction in severity and frequency.

No response: failure to meet the above criteria.

## 3. Results

Our study included 5 patients undergoing various surgical procedures: urological surgery (n = 2), gynecological surgery (n = 1), and proctologic surgery (n = 2). Baseline characteristics are summarized in Table 1.

**Table 1:** Baseline data of this study.

No	Gender	Age	ASA	BMI	LMA	History of hiccups	Doignose	Name of surgery
–	–	Year	–	kg/m^2^	size	–	–	–
1	Female	38	II	25.39	3	None	Abnormal uterine bleeding	Hysteroscopic examination + polyp resection + fractional curettage
2	Male	33	II	35.92	5	None	Ureteral calculi (left)	Ureteroscopic lithotripsy and stone extraction + double-J-stent placement
3	Female	32	II	20.83	3	Yes	Mixed hemorrhoid	Mixed hemorrhoidectomy + partial resection of internal anal sphincter
4	Male	46	II	21.48	4	None	Hematuria (etiology to be determined)	Right renal pelvis holmium laser lithotripsy via ureteroscope + double-J-stent placement
5	Female	27	I	19.04	3	None	Mixed hemorrhoid	Mixed hemorrhoidectomy + internal hemorrhoidal ligation + lateral incision of internal anal sphincter + hypertrophied anal papilla excision

ASA = American Society of Anesthesiologists, BMI = body mass index, LMA = laryngeal mask airway.

### 3.1. Case 1

“Hysteroscopic examination + polyp resection + fractional curettage” were performed under GA. Anesthesia induction: intravenous injection of oxycodone 0.1mg·kg^-1^, remazolam 0.2 to 0.3mg·kg^-1^, intravenous injection of rocuronium 0.2mg·kg^-1^ after BIS < 60, a size 3 disposable SaCo visual LMA(Zhejiang Youyi Medical Equipment Co., Ltd., China) was inserted. Persistent hiccups occurred immediately after LMA cuff inflation, with a frequency of 5 to 7 times·min^-1^, lasting > 3 minutes. Bilateral BL2 acupuncture (technique as described previously) was administered, resulting in hiccups cessation within 5 minutes. The surgery lasted 25 minutes without further hiccup recurrence. The BIS index was 52 before acupuncture and 58 after hiccups cessation, with a fluctuation value of 6, indicating no significant fluctuation. The MAP was 78 mm Hg before acupuncture and 75 mm Hg after hiccups cessation, with a fluctuation of 3 mm Hg, indicating no significant change. No postoperative hiccup recurrence was observed during 24-hour follow-up. The outcome was classified as complete remission.

Cases 2 to 5 received the same anesthetic protocol, LMA type, and acupuncture intervention as case 1.

### 3.2. Case 2

“Ureteroscopic lithotripsy and stone extraction + double-J stent placement” were performed. Hiccups occurred immediately upon initiating positive-pressure ventilation following LMA insertion. with a frequency of 4 to 8 times·min^-1^, lasting > 3 minutes. At 3 minutes post-acupuncture, hiccups frequency decreased to 1 to 3 times·min^-1^, and complete cessation was achieved at 6 minutes. BIS fluctuation was 3 (BIS index: 52 before acupuncture → 55 after hiccups cessation). MAP fluctuation was 5 mm Hg (MAP: 99 before acupuncture → 94 after hiccups cessation).

### 3.3. Case 3

“Mixed hemorrhoidectomy + partial resection of internal anal sphincter” were performed. Hiccups occurred following LMA insertion, with a frequency of 3 to 6 times·min^-1^, lasting > 3 minutes. At 2 minutes post-acupuncture, hiccup frequency decreased to 1 to 2 times·min^-1^, and complete cessation was achieved at 8 minutes. BIS fluctuation: 2 (BIS index: 56 before acupuncture → 58 after hiccups cessation). MAP fluctuation: 4 mm Hg (MAP: 90 before acupuncture → 86 after hiccups cessation).

### 3.4. Case 4

“Right renal pelvis holmium laser lithotripsy via ureteroscope + double J-stent placement” were performed. Hiccups developed 1 minute following LMA insertion, with a frequency of 5 to 8 times·min^−1^, lasting > 3 minutes. At 3 minutes post-acupuncture, hiccup frequency decreased to 0 to 1 times·min^−1^, and complete cessation occurred at 6 minutes. BIS fluctuation: 3 (BIS index: 55 before acupuncture → 52 after hiccups cessation). MAP fluctuation: 4 mm Hg (MAP: 82 before acupuncture → 78 after hiccups cessation).

### 3.5. Case 5

“Mixed hemorrhoidectomy + internal hemorrhoidal ligation + lateral incision of internal anal sphincter + hypertrophied anal papilla excision” were performed. Hiccups occurred following LMA insertion, with a frequency of 3 to 7 times·min^-1^, lasting >3 minutes. Complete cessation was achieved at 4 minutes post-acupuncture. BIS fluctuation: 2 (BIS index: 56 before acupuncture → 58 after hiccups cessation). MAP fluctuation: 3 mm Hg (MAP: 74 before acupuncture → 78 after hiccups cessation).

## 4. Summary

None of the 5 cases experienced hiccups recurrence from cessation until surgery completion. Case 3 developed a single postoperative hiccup recurrence 30 minutes after surgery. Bilateral BL2 acupoints were compressed sequentially using the thumb pulp of both hands, with gradually increasing pressure until the patient reported tolerable soreness and distension. The intervention involved alternating 10-second cycles of compression and release. Hiccups ceased within 3 minutes, with no recurrence observed during postoperative follow-up at 24 hours. The therapeutic outcome was categorized as partial remission. The remaining cases showed no recurrence within 24 hours postoperatively, meeting criteria for complete remission. None of the 5 cases exhibited local acupuncture-related pain (VAS = 0), hemorrhage, or ecchymosis. All cases had no awareness during the acupuncture treatment and experienced no discomfort following the procedure. Both BIS and MAP remained stable before intervention and after hiccups cessation (Table 2).

**Table 2:** Validity of the study.

No	Hiccups frequency before intervention (times·min^-1^)	The termination time of hiccups after acupuncture (min)	Cessation pattern	Number of recurrence/cumulative (time)	Adverse events pain (VAS)/hemorrhage/ecchymosis	Fluctuation of BIS	Fluctuation of MAP (mm Hg)	Operation time (min)	Outcome
1	5–7	5	abrupt	0/0	0/None/None	6	3	25	Complete remission
2	4–8	6	gradual	0/0	0/None/None	3	5	15	Complete remission
3	3–6	8	gradual	1/3	0/None/None	2	4	15	Partial remission
4	5–8	6	gradual	0/0	0/None/None	3	4	60	Complete remission
5	3–7	4	abrupt	0/0	0/None/None	2	4	20	Complete remission

BIS = bispectral index, MAP = mean arterial pressure, VAS = visual analogue scale.

## 5. Discussion

Hiccups are involuntary myoclonic contractions of the diaphragm and intercostal muscles, resulting in sudden inspiration followed by abrupt glottic closure.^[9]^ The underlying mechanisms remain incompletely understood, though prevailing theories posit that hiccups constitute a complex neural reflex activity. Sensory fibers of the vagus, phrenic, and T6 to 12 sympathetic nerves transmit neural impulses to central systems (hypothalamus, brainstem, C3 to 5 cervical spinal cord pons), which are relayed via efferent pathways through the phrenic and intercostal nerves, inducing coordinated contractions of intercostal muscles, glottis, and diaphragm.^[10]^ This reflex arc comprises afferent pathways, central neural integration, and efferent pathways,^[11]^ any structural or functional perturbation affecting this reflex arc may precipitate hiccups.^[9]^ Patients with a history of intractable or persistent hiccups demonstrate increased susceptibility to perioperative recurrence, even in the absence of preoperative symptoms, due to anesthetic and surgical influences.^[11]^ Furthermore, patients without inherent predisposition may experience elevated perioperative hiccup incidence when undergoing interventions proximate to the anatomical course of the hiccup reflex arc.

Perioperative hiccup triggers encompass surgical manipulation, anesthetic factors, and underlying patient comorbidities.^[11]^ In the reported cases, the BIS index was maintained between 40 and 60, indicating adequate depth of anesthesia, making inadequate anesthesia depth an unlikely contributor.

The onset of hiccups occurred following LMA insertion prior to surgical commencement, suggesting potential associations with anesthetic agents or techniques employed. Anesthetic interventions such as mask ventilation-induced gastric distension and pharmacological effects of induction agents (e.g., opioids, remimazolam) may induce hiccups.^[1,11–14]^ The LMA itself can also induce hiccups, with an incidence ranging from 1% to 14%.^[15]^ The vagus nerve innervates both pharyngeal and proximal esophageal regions. It is hypothesized that stimulation of the vagus nerve during LMA insertion or rapid cuff inflation may serve as a triggering factor for hiccups.^[1]^ This phenomenon may occur when the LMA tip is positioned near the proximal esophagus, thereby activating local mechanoreceptors and triggering vagal excitation.^[16]^

Perioperative hiccups may induce sudden involuntary patient movement, potentially delaying surgical procedures, compromising intraoperative monitoring and respiratory function, interfering with procedural accuracy, and elevating the risk of complications. In severe cases, they may necessitate suspension or termination of the procedure.^[11,17]^ Hiccups further increase the aspiration risk by generating pressure gradients across the lower esophageal sphincter.^[18]^ Prompt management of perioperative hiccups is therefore critical to avoiding these potential complications. Therapeutic strategies encompass pharmacological and non-pharmacological approaches. Chlorpromazine remains the sole FDA-approved pharmacological intervention for hiccups; however, it may cause hypotension and sedation, which could compromise hemodynamic stability and interfere with the assessment of anesthetic depth.^[3]^ Therefore, its intraoperative use thus requires caution.

Due to the potential adverse effects of medications, non-pharmacologic alternatives (e.g., stellate ganglion block, phrenic neuromodulation, and acupuncture) have been explored.^[19–21]^ Among these, acupuncture has garnered growing interest.^[22,23]^ Compared to pharmacological and other therapies, acupuncture demonstrates superior efficacy in treating hiccups with a lower incidence of adverse effects and a more favorable safety profile.^[24,25]^

Previous studies have validated the effectiveness of acupuncture combined with acupoint injection therapy for persistent hiccups of peripheral origin,and acupuncture at Siguan points has also proven effective in treating hiccups associated with painless gastroscopy.^[17,22]^ Commonly employed acupoints, such as Neiguan (PC6), Zusanli (ST36), BL2, and Geshu (BL17), have been documented in the management of non-perioperative or postoperative hiccups;^[17,22,26,27]^ while perioperative applications remain unreported. This case series is the first to report the successful use of intraoperative acupuncture at BL2 for managing hiccups under GA, offering a new non-pharmacological approach to this clinical challenge.

The management of intraoperative hiccups poses significant challenges in surgeries with specific requirements. For instance, procedures requiring neuromonitoring should limit or avoid NMBA,^[28]^ while those necessitating intraoperative wakefulness demand precise control of anesthetic depth and drug dosage. In such cases, non-pharmacological interventions (e.g., acupuncture) should be prioritized.

However, due to intraoperative aseptic protocols, the patient’s trunk and limbs are often covered with sterile drapes, making it difficult to expose and access routinely used acupoints for hiccup treatment, such as ST36, PC6, and BL17. In contrast, BL2, located at the supraorbital notch on the face, remains uncovered outside the sterile drapes in most procedures (except head and facial surgeries), allowing for easier manipulation. BL2 acupuncture combined with PC6 and ST36 achieved a 93.18% efficacy rate for post-cranio-cerebral trauma hiccups (vs 69.05% in controls).^[28]^ BL2 and CV17 acupuncture with metoclopramide acupoint injection proved safe and effective for persistent hiccups.^[22]^

Previous investigations have validated the clinical efficacy of BL2 stimulation for managing non-perioperative hiccups.^[29]^ This study extends these findings to the intraoperative setting, demonstrating complete hiccup cessation within 4 to 8 minutes post-intervention in all 5 cases. Notably, Cases 2 and 4 exhibited a reduced hiccup frequency at 3 minutes post-needling, while Case 3 showed a frequency reduction at 2 minutes, with complete cessation achieved by 8 minutes. In contrast, Cases 1 and 5 displayed abrupt termination without preceding frequency reduction. This difference in gradual versus sudden termination may reflect distinct neuromodulatory mechanisms – Progressive cases suggest cumulative parasympathetic nervous system activation, abrupt cessation may indicate immediate diaphragmatic movement inhibition via supratrochlear nerve stimulation.

In Case 3, hiccups recurred 30 minutes postoperatively but were resolved after applying pressure to bilateral BL2 for 3 minutes, with no further recurrence during the 24-hour postoperative follow-up. This patient had a history of gastroesophageal reflux disease, and hiccups had previously been triggered by eating too quickly, exposure to cold, or emotional agitation – factors that may explain the recurrence after laryngeal mask removal upon emergence from GA. The other 4 cases showed no recurrence of hiccups within 24 hours postoperatively. Upon further inquiry, these patients had no history of central or peripheral conditions predisposing them to hiccups, nor had they experienced chronic or refractory episodes in the past.

The therapeutic effect of acupuncture at BL2 may be attributed to its unique neuroanatomical characteristics and its modulatory effect on central reflexes. Anatomically, the superficial layer of BL2 contains the frontal nerve (a branch of the ophthalmic division of the trigeminal nerve), while deeper layers involve branches of the facial nerve and supratrochlear nerve. Strong stimulation of these nerves may directly excite higher cortical centers, thereby suppressing abnormal vagal nerve excitation and terminating hiccups.^[30]^ The recurrence and subsequent rapid relief of postoperative hiccups in Case 3 through BL2 pressure suggests this acupoint may exert a dual regulatory effect, modulating both peripheral afferent pathways and central processing mechanisms.

Acupuncture may locally modulate the hiccup reflex arc through multiple mechanisms, including changes in blood perfusion, autonomic nervous system activation, inflammatory mediator modulation, and axonal excitability regulation. Furthermore, by stimulating peripheral nerve endings at the acupoint, acupuncture promotes the synthesis and release of neurotransmitters such as norepinephrine, serotonin, and γ-aminobutyric acid, thereby inhibiting hiccup generation.^[31,32]^

No local pain, hematoma, infection, or other acupuncture-related complications were observed in any of the 5 cases, which aligns with the safety profile of acupuncture reported in previous studies.^[32]^

The BIS index and MAP demonstrated no significant fluctuations before acupuncture at BL2 or after the termination of hiccups, indicating that this intervention had negligible impacts on anesthetic depth or hemodynamic stability. The immediate cessation of hiccups in Cases 1 and 5, in contrast to the gradual resolution pattern observed in Cases 2 to 4, suggests the necessity for further stratified analysis. Additionally, the comparative efficacy between single-acupoint intervention and multi-acupoint combination therapies warrants further investigation.

### 5.1. Limitations

This small case series (n = 5) necessitates larger validation studies to exclude chance outcomes. Unable to distinguish between natural relief and acupuncture effects, randomized controlled trials (RCTS) can be designed in the future. The lack of blinding in the assessment of the primary outcome (hiccup cessation) presents a potential for observer bias. Heterogeneous etiologies and anesthetic regimens may influence treatment response uniformity. Retrospective data collection risks confounding factor omission (e.g., laryngeal mask pressure, patient positioning), and prospective data collection is needed.

## 6. Conclusion

The case series suggests acupuncture at BL2 may be effective in terminating intraoperative hiccups, though controlled trials are needed to confirm this preliminary finding. No adverse events (such as local pain, hemorrhage or ecchymosis) were observed in any case, and stable BIS and MAP during the intervention confirmed its favorable tolerability. As a non-pharmacological intervention, this technique is particularly suitable for high-risk surgical patients with contraindications to neuromuscular blocking agents or those requiring avoidance of pharmacological adverse effects. Further research is warranted to elucidate its mechanisms and therapeutic efficacy.

## Author contributions

**Conceptualization:** Li Chen, Guo-Ao Shi.

**Data curation:** Guo-Ao Shi, Xu Dong, Chun-Ru Zhang.

**Formal analysis:** Shan Wang, Chun-Ru Zhang, Dong-Sen Lv.

**Investigation:** Guo-Ao Shi, Xu Dong, Li Kang.

**Methodology:** Li Chen, Shan Wang, Dong-Sen Lv.

**Supervision:** Li Chen, Dong-Sen Lv.

**Writing – original draft:** Li Chen, Shan Wang.

**Writing – review & editing:** Guo-Ao Shi, Xu Dong.
